# Non‐steroidal anti‐inflammatory drugs, prostaglandins, and COVID‐19

**DOI:** 10.1111/bph.15206

**Published:** 2020-08-27

**Authors:** Calum T. Robb, Marie Goepp, Adriano G. Rossi, Chengcan Yao

**Affiliations:** ^1^ Centre for Inflammation Research, Queen's Medical Research Institute The University of Edinburgh Edinburgh UK

**Keywords:** COVID‐19, cytokine storm, inflammation, NSAID, prostaglandin, SARS‐CoV‐2

## Abstract

Severe acute respiratory syndrome coronavirus 2 (SARS‐CoV‐2) is the cause of the novel coronavirus disease 2019 (COVID‐19), a highly pathogenic and sometimes fatal respiratory disease responsible for the current 2020 global pandemic. Presently, there remains no effective vaccine or efficient treatment strategies against COVID‐19. Non‐steroidal anti‐inflammatory drugs (NSAIDs) are medicines very widely used to alleviate fever, pain, and inflammation (common symptoms of COVID‐19 patients) through effectively blocking production of prostaglandins (PGs) via inhibition of cyclooxyganase enzymes. PGs can exert either proinflammatory or anti‐inflammatory effects depending on the inflammatory scenario. In this review, we survey the potential roles that NSAIDs and PGs may play during SARS‐CoV‐2 infection and the development and progression of COVID‐19.

**Linked Articles:**

This article is part of a themed issue on The Pharmacology of COVID‐19. To view the other articles in this section visit http://onlinelibrary.wiley.com/doi/10.1111/bph.v177.21/issuetoc

**Abbreviations**
AA: arachidonic acid
ACE2: angiotensin‐converting enzyme 2
ALI: acute lung injury
ARDS: acute respiratory distress syndrome
AT1: type‐I pneumocyte
AT2: type‐II pneumocyte
BAL: bronchoalveolar lavage
CI: confidence interval
COPD: chronic obstructive pulmonary disease
CoV: coronavirus
COVID‐19: coronavirus disease 2019
COX: cyclooxygenase
DC: dendritic cell
IAV: influenza A virus
IL: interleukin
IRF: IFN regulatory factor
ISG: IFN‐stimulated genes
MERS: Middle East respiratory syndrome
mPGES: microsomal PGE synthase
NETs: neutrophil extracellular traps
NLRP3: NOD‐, LRR‐, and pyrin domain‐containing protein 3
NSAID: non‐steroidal anti‐inflammatory drug
Nsp: non‐structural proteins
OR: odds ratio
ORF: open reading frame
PG: prostaglandin
PGE_2_: prostaglandin E_2_
PTGES: PGE synthase
RSV: respiratory syncytial virus
SAA: serum amyloid A
SARS‐CoV‐1: severe acute respiratory syndrome coronavirus 1
SARS‐CoV‐2: severe acute respiratory syndrome coronavirus 2
Th1: type 1 helper T cell
Th17: type 17 helper T cell
TLR: toll‐like receptor
TMPRSS2: transmembrane protease serine 2
Treg: regulatory T cell

## 1 INTRODUCTION

The novel coronavirus disease 2019 (COVID‐19) was first reported in late 2019, Wuhan, China, and is now a global pandemic with >12 million confirmed cases and more than half a million deaths in over 200 countries and territories encompassing Africa, Americas, Eastern Mediterranean, Europe, South East Asia, and the Western Pacific (World Health Organization, COVID‐19 Situation Report 175, July 13, 2020). COVID‐19 is characterised by cough, fever, and shortness of breath confirmed by a study of 20,133 hospitalised UK patients (Docherty et al., 2020). COVID‐19 causes a spectrum of respiratory conditions differing in severity, including mild upper respiratory tract complications, acute respiratory distress syndrome (ARDS), and pneumonia (due to secondary infection) in more deleterious cases. Evidence from meta and genetic analyses indicates that co‐morbidities including chronic cardiac/kidney disease, non‐asthmatic chronic pulmonary disease, chronic obstructive pulmonary disease (COPD), cardiovascular disease, diabetes, liver disease, obesity, hypertension, and cerebrovascular disease are associated with higher incidence of mortality in COVID‐19 patients (Docherty et al., 2020; Wang, Li, Lu, & Huang, 2020). Furthermore, post‐mortem examinations reveal that tissue inflammation and organ dysfunction do not map to tissue/cellular dispersal of severe acute respiratory syndrome coronavirus 2 (SARS‐CoV‐2) during fatal COVID‐19 (Dorward et al., 2020). Thus it was concluded that immune‐mediated as opposed to pathogen‐mediated organ inflammation and injury contributed to death in COVID‐19 patients (Dorward et al., 2020).

COVID‐19 is caused by SARS‐CoV‐2, which generally has a 4‐ to 5‐day average incubation period (prior to onset of symptoms), with 97.5% of symptomatic patients experiencing symptoms within 11.5 days (Lauer et al., 2020), although patients can also be asymptomatic. SARS‐CoV‐2 is normally transmitted via respiratory droplets to facial mucosal membranes (eyes, nose, and mouth) with high efficacy over short distances. SARS‐CoV‐2 belongs to a family of viruses named *Coronaviridae*, which are a broad range of single‐stranded, positive‐sense, RNA viruses that can cause respiratory, enteric, neurological, and hepatic diseases in multiple animal species and humans (Zumla, Chan, Azhar, Hui, & Yuen, 2016). α‐Coronaviruses (αCoVs, e.g., HCoV‐229E and HCoV‐NL63) and β‐coronaviruses (βCoVs, e.g., HCoV‐OC43 and HCoV‐HKU1) are two of four *Coronaviridae* genres which commonly infect humans (Liu, Liang, & Fung, 2020). These HCoVs usually cause mild to moderate upper respiratory tract infections, for example, the common cold.

However, over the last 18 years, three novel βCoVs have emerged causing three separate deadly respiratory disease outbreaks in humans. These novel βCoVs include severe acute respiratory syndrome coronavirus 1 (SARS‐CoV‐1) first identified in 2003, Guangdong, China; Middle East respiratory syndrome coronavirus (MERS‐CoV) first identified in 2012, Jeddah, Saudi Arabia; and most recently, SARS‐CoV‐2 (N. Chen, Zhou, et al., 2020; Khan et al., 2020). SARS‐CoV‐2 has caused far greater infectivity and transmission potential compared to SARS‐CoV‐1 and MERS‐CoV. SARS‐CoV‐2 shares roughly 79% and 50% genetic similarity with SARS‐CoV‐1 and MERS‐CoV, respectively (Lu et al., 2020), but is strikingly 96.1% genetically similar to bat CoV‐RaTG13 (H. Zhou, Chen, et al., 2020). This relatively high genetic similarity with SARS‐CoV‐1 suggests that SARS‐CoV‐2 may exert its pathogenesis via similar mechanisms.

Pathophysiology of SARS‐CoV‐2 culminates in airway injury (possibly by direct effects on epithelial cells) and powerful host inflammatory responses (e.g., neutrophilic and macrophage inflammation and cytokine storm), as previously observed in SARS‐CoV‐1 (G. Chen, Wu, et al., 2020; Huang et al., 2020). Hence, both viral infection and host responses contribute to disease severity. Regarding epidemiology, the correlation between increasing severity with age of infected SARS‐CoV‐2 patients does correspond with epidemiology of SARS‐CoV‐1 and MERS‐CoV (Tay, Poh, Rénia, MacAry, & Ng, 2020). SARS‐CoV‐1 infects various lung cells including ciliated airway epithelial cells, vascular endothelial cells, macrophages, and type II pneumocytes (AT2 cells) via binding to the host cell receptor, angiotensin‐converting enzyme 2 (ACE2), and the transmembrane protease serine 2 (TMPRSS2) for S protein priming (Cui, Li, & Shi, 2019; Hamming et al., 2004; Jia et al., 2005; Qian et al., 2013; H. Xu, Zhong, et al., 2020). Conversely, MERS‐CoV infects unciliated airway epithelial cells as well as type I (AT1 cells) and AT2 cells, via dipeptidyl peptidase‐4 (DPP‐4; CD26) (Cui et al., 2019; de Wit et al., 2013; Raj et al., 2013). As the infection route of SARS‐CoV‐2 is also via the ACE2 receptor and TMPRSS2, it is expected that lung cells are infected as defined in SARS‐CoV‐1 (Hoffmann et al., 2020; Walls et al., 2020; Zhao et al., 2020; P. Zhou, Yang, et al., 2020). Moreover, as ACE2 expression in lung cells is lowered during SARS‐CoV‐1 infection and loss of ACE2 function has been shown to contribute to acute lung injury (ALI) (Imai, Kuba, & Penninger, 2008; Imai et al., 2005; Kuba et al., 2005; Kuba, Imai, Rao, Jiang, & Penninger, 2006), a reduction in ACE2 receptor expression may be a key mediator of COVID‐19 pathogenesis, featured as hyperinflammation and thrombosis. So far, there remains no clear antiviral treatments or vaccine against COVID‐19.

### 2 NSAIDs AND COVID‐19: FRIENDS OR FOES?

Although infection with SARS‐CoV‐2 can be asymptotic, the most common clinical symptoms for COVID‐19 usually start from fever, dry cough, fatigue, muscle pain, dyspnoea, anosmia, and ageusia days after catching the virus. Disease in some patients can then progress to severe/critical conditions and which regularly results in death. The risk factors for severe/critical disease include age, male sex, and co‐morbidities, but young COVID‐19 patients without underlying medical conditions can also develop severe disease (Huang et al., 2020; Oxley et al., 2020; Williamson et al., 2020). Compared to surviving patients, non‐surviving COVID‐19 patients had gradually and remarkably elevated inflammatory mediators in their blood, such as C‐reactive protein, D‐dimer, cytokines, for example, IL‐6, IL‐8, and TNF‐α, and neutrophil‐to‐lymphocyte ratio, progressing to ARDS, sepsis, and multi‐organ failure (D. Zhang, Guo, et al., 2020; F. Zhou, Yu, et al., 2020). This observation was confirmed by a systemic proteomic analysis of COVID‐19 patient sera (Shen et al., 2020), indicating a key role for hyperinflammation in COVID‐19 progression. Targeting inflammation is thus one of key strategies to manage COVID‐19 disease severity.

Non‐steroidal anti‐inflammatory drugs (NSAIDs), such as aspirin, ibuprofen, celecoxib, and indomethacin, are a group of medicines that are used very widely from managing acute (e.g., fever and pain) and chronic (e.g., rheumatoid arthritis) inflammatory conditions to preventing and/or treating cancers. NSAIDs are commonly used by COVID‐19 patients to reduce fever and relieve muscle pain, but whether NSAIDs are beneficial or harmful to COVID‐19 patients has become a hot topic. The use of NSAIDs thus far during the COVID‐19 pandemic remains controversial, and a cautionary approach is advised (Day, 2020; FitzGerald, 2020; Little, 2020). Indeed, NSAIDs are more likely to be regularly used by elderly people (>60 years) with existing health conditions such as high body mass index, increased waist circumference, or heart disease (Davis et al., 2017), which collectively represent risk factors for COVID‐19. Although the World Health Organization and the United Kingdom National Health Service have declared that there is no evidence of an increased risk of developing COVID‐19 or making the disease more severe by using NSAIDs, suggestions on avoiding NSAIDs for COVID‐19 patients were previously made based on numerous clinical trials and observations on non‐COVID‐19 pulmonary infectious diseases. For example, apart from the commonly adverse effects of NSAIDs such as gastrointestinal, renal, and cardiovascular complications (Bhala et al., 2013; Little et al., 2013), NSAIDs were found to cause more prolonged illness or complications when taken during respiratory tract infections (Le Bourgeois et al., 2016; Voiriot et al., 2019). It was reported that use of NSAIDs for fever or non‐rheumatologic pain during the early stages of infection increased the risk of severe bacterial superinfection (Micallef, Soeiro, & Jonville‐Béra, 2020). NSAIDs may increase hypercoagulation and the incidence of thrombosis due to decreased thrombomodulin (Rabausch et al., 2005; Schmidt et al., 2011), a particular concern given that COVID‐19 patients often have coagulation abnormalities and increased vascular clotting (Levi, Thachil, Iba, & Levy, 2020). It is plausible that NSAIDs may possibly inhibit protective host immune reactions against coronavirus replication (Wu et al., 2020) and enhance the proinflammatory cytokine storm observed in lungs of COVID‐19 patients, for example, through activation of inflammatory macrophages (Wu & Meydani, 2008). Moreover, as SARS‐CoV‐2 can infect human gut enterocytes (Lamers et al., 2020), it is important to know if NSAIDs synergise with SARS‐CoV‐2 infection to potentiate severe intestinal damage.

However, do NSAIDs have any potential beneficial effects on the risk of infection with SARS‐CoV‐2 and/or COVID‐19 severity? In a retrospective cohort study, Rentsch et al. (2020) found that exposure to NSAIDs (−365 to −14 days prior to baseline) was modestly associated with increased likelihood of COVID‐19 infection (multivariable odds ratio [OR] 1.27, 95% confidence interval [CI] 1.02–1.58), was not associated with hospitalisation (*P* = 0.19), and had a negative trend of association with intensive care (*P* = 0.08). In this study, information of NSAIDs only obtained from pharmacy records was counted, but individuals assigned as non‐NSAID users were still likely to get NSAIDs over the counter. In another study, Freites et al. (2020) found that use of NSAIDs was associated with reduced risk of hospital admission of COVID‐19 patients who had chronic inflammatory rheumatic disease (OR 0.37, 95% CI 0.15–0.91, *P* = 0.03). Castro, Ross, McBride, and Perlis (2020) reported that prescription of ibuprofen and naproxen (both NSAIDs) was also associated with reduced hospitalisation among 2,271 individuals who tested positive for COVID‐19 (OR 0.649, 95% CI 0.415–0.992 and OR 0.388, 95% CI 0.144–0.930, respectively) after adjustments for age, sex, race, ethnicity, site, and combined co‐morbidity index. Among hospitalised COVID‐19‐positive individuals, 29 out of 34 patients premedicated with ibuprofen (adjusted OR 0.421, 95% CI 0.139–1.046) and 6 out of 7 patients premedicated with naproxen (adjusted OR 0.362, 95% CI 0.019–2.269) did not require mechanical ventilation during hospitalisation (Castro et al., 2020). Huh et al. performed a large case–control study assessing the relationships of the risk of COVID‐19 and the use of various drugs in 65,149 adult subjects (>18 years old, 7.9% were tested COVID‐19 positive) using a national wide claims database of South Korea. They found that prior exposure to aspirin significantly associated with reduced risk of developing COVID‐19 (adjust OR 0.29, 95% CI 0.14–0.58, *P* < 0.001), while prior use of COX‐2 inhibitors did not significantly reduce the risk (Huh et al., 2020). Interestingly, Hong et al. (2020) have recently performed a pilot trial to treat COVID‐19 patients with celecoxib (Celebrex ®; a selective inhibitor of COX‐2) at either 0.2 g twice or once a day. The remission rates of COVID‐19 disease for full/high (0.2 g twice a day) and half/medium (0.2 g once a day) doses of celecoxib and control groups were 100%, 82%, and 57%, respectively (Hong et al., 2020). Celecoxib treatment also improved pulmonary opacification and pneumonia faster than control group based on chest CT scan results. This study suggested that celecoxib may promote the recovery of ordinary and severe cases of COVID‐19 and prevent the progression of severe disease to a critical stage (Hong et al., 2020). Low doses of celecoxib (i.e., 0.2 g once a day) used in this study was associated with a small increase in the risk (relative risk 1.35, 95% CI 1.00–1.82) of non‐fatal myocardial infarction, but the high dose of celecoxib (i.e., 0.2 g twice a day) was not associated with increased risk of myocardial infarction (relative risk 1.05, 95% CI 0.33–3.35) (García Rodríguez, Tacconelli, & Patrignani, 2008). These findings may thus be indicative of the safety and efficiency for use of such doses of celecoxib in COVID‐19 patients who have known cardiovascular conditions.

These (pharma)epidemiological and clinical observations suggest that NSAIDs may be beneficial in controlling the development of severe COVID‐19. The types and doses of NSAIDs determine their capability of inhibiting enzymic activities of COX‐1 or COX‐2 or both, which affects the production profile of downstream eicosanoids, resulting in varied side effects in the cardiovascular, gastrointestinal, renal, and other systems (Bhala et al., 2013; García Rodríguez et al., 2008; Little et al., 2013). However, the information for exposure of NSAIDs (e.g., drug types, doses, and exposure time) in the above epidemiological studies was unclear as they were mainly defined based on electronic health records. Lack of such information impedes further assessment of clinical benefits or harm of NSAIDs. Therefore, large‐scale, double‐blinded, randomised, and well‐controlled clinical trials are warranted to thoroughly assess the effects of specific NSAIDs at appropriate doses in prevention and treatment of COVID‐19 while also attempting to avoid their known side effects.

Moreover, another NSAID, indomethacin, has shown potential antiviral activity against human SARS‐CoV‐1 and canine coronavirus (Amici et al., 2006). Indomethacin does not affect coronavirus binding or entry into host cells but instead acts by blocking viral RNA synthesis in vitro. Oral administration of indomethacin (1 mg·kg^−1^·day^−1^ [equivalent ~40 mg·kg^−1^ for a 70‐kg human, less than the low‐medium dose of 75 mg daily used in adult patients] for 4 days starting on day 4 post‐infection) markedly reduced (by ~2–3 orders) shedding of canine CoV RNA in the faeces of dogs infected with canine CoV, but this antiviral effect was reversed upon suspension of indomethacin treatment (T. Xu, Gao, Wu, Selinger, & Zhou, 2020). Indomethacin has also been suggested to exhibit potent antiviral activity against SARS‐CoV‐2‐infected Vero E6 cells in vitro and canine CoV‐infected dogs in vivo (T. Xu, Gao, et al., 2020). A very recent study based on a multi‐stage model‐based approach showed that treatment with the sustained‐release formulation of indomethacin at the dose of 75 mg twice a day (high dose used clinically according to García Rodríguez et al., 2008) is expected to achieve a complete response in 3 days for the treatment in patients infected by SARS‐CoV‐2, suggesting that indomethacin could be considered as a promising candidate for the treatment of COVID‐19 (Gomeni, Xu, Gao, & Bressolle‐Gomeni, 2020; Koch et al., 2020). The antiviral capacity of indomethacin is conferred by activation of protein kinase R, independently of interferons and double‐stranded RNA (Amici et al., 2015) but may be via interactions with aldoketo‐reductases, aldose reductases, PPAR‐γ, and the cannabinoid CB_2_ receptor (T. Xu, Gao, et al., 2020). Conversely, it is important to report that indomethacin and other NSAIDs (including coxibs) are associated with nephrotoxicity (Delaney & Segel, 1985; McCarthy, Torres, Romero, Wochos, & Velosa, 1982; Whelton, 1999). As a result, such NSAIDs are not recommended for use in patients with clinically complicated SARS‐CoV‐2 infections with deficiencies in renal function, where renal blood flow is maintained by the contributory effects of vasodilator PGs (Grosser, Fries, & FitzGerald, 2006) or with gastrointestinal risk factors (Capuano et al., 2020). Therefore, such clinically complex SARS‐CoV‐2 infections constitute a contraindication to the use of NSAIDs as their use may predispose to these well‐known side effects of NSAIDs (Patrignani & Patrono, 2015). Importantly, indomethacin is also known to have coronary vasoconstrictor effects via blockade of vasodilatory PG synthesis or to a direct drug effect, suggesting that in patients with severe coronary‐artery disease, indomethacin should be used with caution (Friedman et al., 1981). Using an original virtual screening protocol, celecoxib at 50 μM (much higher than its maximum serum concentration of 1.8 μM when 200 mg·day^−1^ is used) was predicted to suppress the activity of the main chymotrypsin‐like protease (a key target for antiviral drugs) of SARS‐CoV‐2 by ~12% (Gimeno et al., 2020). In addition, naproxen has also been shown to possess antiviral activity against influenza A and B viruses by interfering with the RNA replication process (Zheng et al., 2019). Moreover, ibuprofen was reported to enhance ACE2 expression from diabetic rat cardiac tissues (Qiao et al., 2015), while celecoxib was shown to repress expression of TMPRSS2 in human prostate cancer cells (Kashiwagi et al., 2014). Given the protective action of ACE2 in ALI (Imai et al., 2008; Imai et al., 2005; Kuba et al., 2005; Kuba et al., 2006) and the negative correlation between ACE2 expression and SARS‐CoV‐2 severe outcomes (J. Chen, Jiang, et al., 2020), NSAIDs may help reduce COVID‐19 disease severity due to SARS‐CoV‐2 infection if they can up‐regulate and down‐regulate ACE2 and TMPRSS2 in the lung, respectively.

## 3 PGs IN THE HUMAN LUNG

**FIGURE 1:**
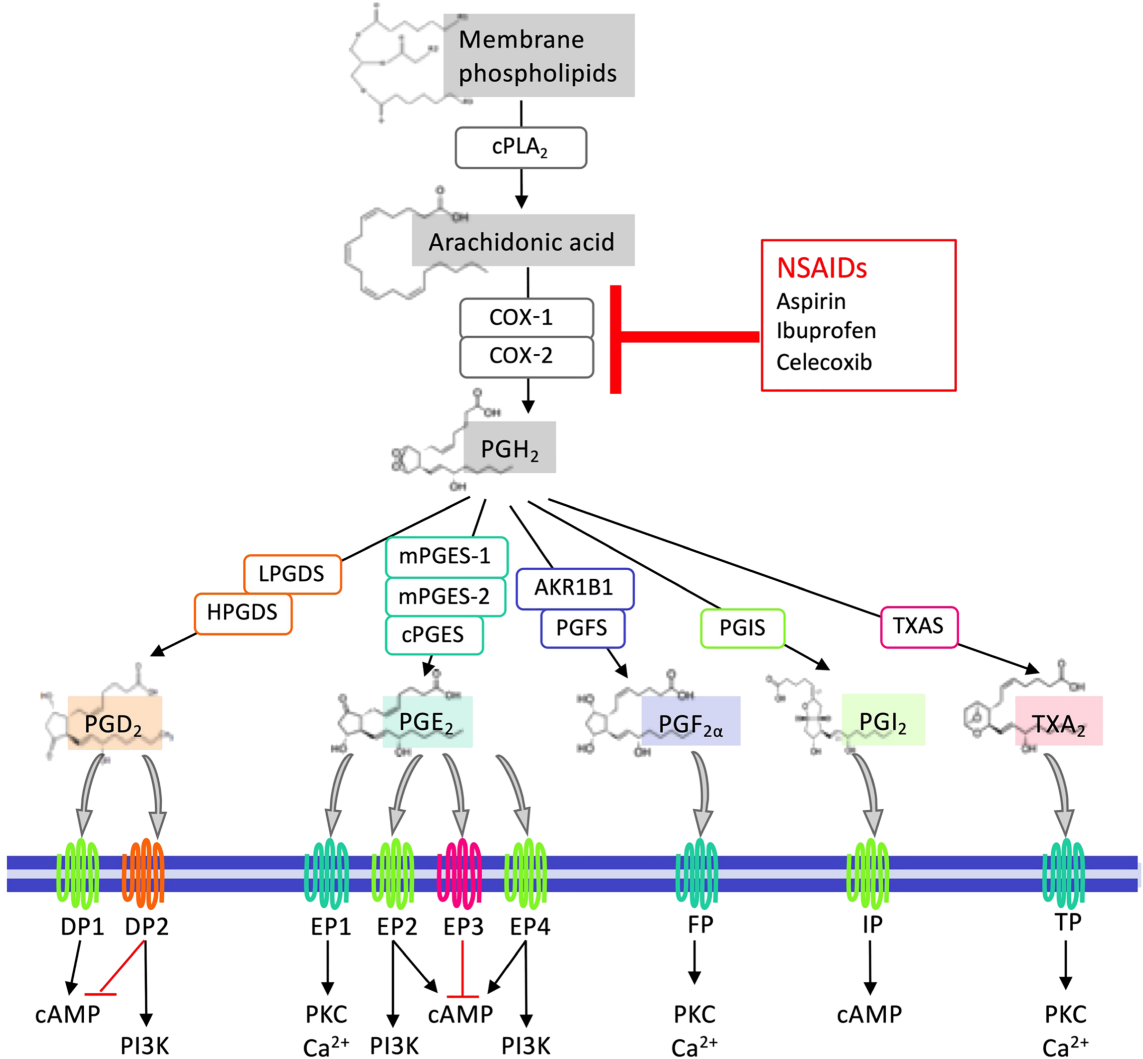
An overview of PG biosynthesis, receptors, and downstream signalling pathways. Arachidonic acid (AA) is released from membrane phospholipids via the actions of cytosolic PLA_2_ (cPLA_2_) following various stimuli and then metabolised to PGH_2_ by COXs (COX‐1 and COX‐2). PGH_2_ is unstable and subsequently converted into PGs, that is, PGD_2_, PGE_2_, PGF_2α_, PGI_2_, and TXA_2_ by the actions of their synthases PGDS (LPGDS and HPGDS), PGES (mPGES‐1, mPGES‐2, and cPGES), PGFS (AKR1B1 and PGFS/ABR1C3), PGIS, and TXAS, respectively. PGs bind to their receptors and activate different downstream signalling pathways. PGD_2_ receptors, DP1 and DP2, activate the cAMP and PI3K pathways, respectively, while DP2 also represses the cAMP pathway. PGE_2_ receptors EP2 and EP4 activate both cAMP and PI3K pathways, EP1 activates PKC and Ca^2+^ pathways, and EP3 deactivates the cAMP pathway. Both PGF_2α_ receptor FP and TXA_2_ receptor TP activate PKC and Ca^2+^ pathways, whereas PGI_2_ receptor IP triggers activation of cAMP signalling. On the other hand, non‐steroidal anti‐inflammatory drugs (NSAIDs) inhibit AA biosynthesis of all PGs by targeting COX‐1 and/or COX‐2

**FIGURE 2:**
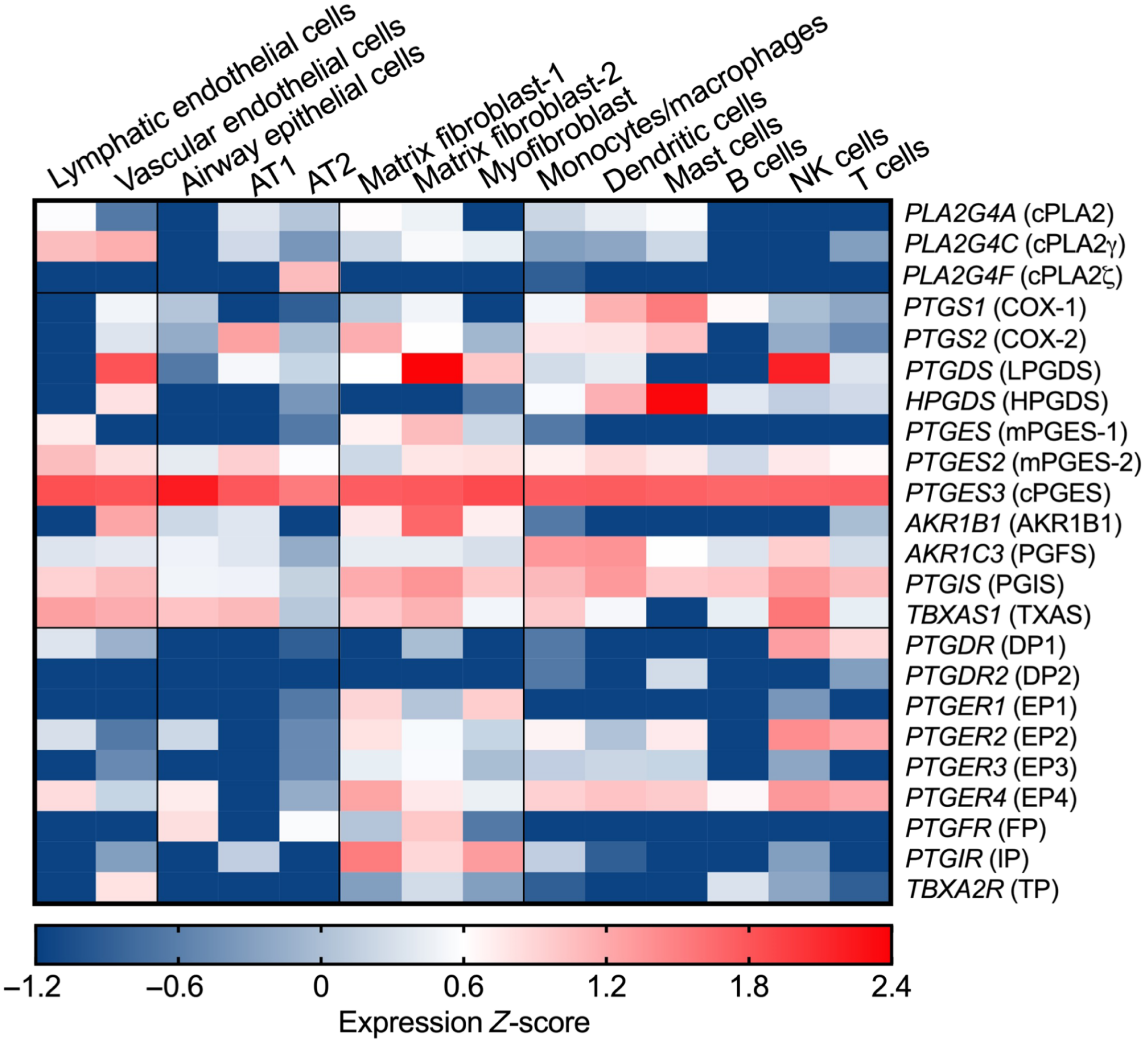
Gene expression profiles of PG synthases and their receptors in human lung cells. Gene expression data from single‐cell RNA sequencing analysis of healthy lung cells from a young individual were retrieved from LungGENS (Lung Gene Expression iN Single‐cell, Du et al., 2017) and transformed to *Z*‐score. AT1, type 1 alveolar epithelial cell (pneumocyte); AT2, type 2 alveolar epithelial cell (pneumocyte). Words in Italic represent human gene symbols, while words in brackets represent human protein names

NSAIDs serve to reduce inflammation by targeting cyclooxygenases (COXs, i.e., COX‐1 and COX‐2) and inhibiting biosynthesis of prostaglandins (PGs), a group of important lipid mediators. PGs are formed when arachidonic acid (AA) is released from cell membrane phospholipids by the actions of cytosolic PLA_2_ (cPLA_2_) and converted to PGH_2_ by COXs. COX‐1 is constitutively expressed in most cells, whereas COX‐2 is induced upon the initiation of inflammation (Dubois et al., 1998). PGH_2_ is unstable and converted into each PG by the corresponding specific synthases. Thus PGD_2_ is produced by the PGD synthases, LPGDS and HPGDS); PGE_2_ by the PGE synthases (mPGES‐1, mPGES‐2, and cPGES); PGF_2α_ by the PGF synthases, including AKR1C3 and AKR1B1; PGI_2_ (prostacyclin) by the PGI synthase: and thromboxin A_2_ (TXA_2_) by TXA synthase (Figure 1). Single‐cell RNA sequencing analysis (Du et al., 2017) reveals that cPLAs, COXs, and PG synthases are expressed in normal human lung cells from a healthy young individual (Figure 2). For example, COX‐1 is expressed in immune cells such as dendritic cells (DCs) and mast cells while COX‐2 is broadly but moderately expressed by epithelial (e.g., AT1), stromal (e.g., matrix fibroblasts), and immune (e.g., monocytes/macrophages, DCs, and mast cells) cells. Endothelial, stromal, mast, DC, and NK cells highly express PGDSs, but only matrix fibroblasts and lymphatic endothelial cells moderately express mPGES‐1, the inducible enzyme that mediates PGE_2_ biosynthesis in vivo, in the normal lung.

PGs exert their autocrine and paracrine effects via activation of nine types and subtypes of rhodopsin‐like transmembrane spanning GPCRs. These are the PGE_2_ receptors (EP1, EP2, EP3 and EP4), PGD_2_ receptors (DP1 and DP2), PGF_2α_ receptor (FP), PGI_2_ receptor (IP), and TXA_2_ receptor (TP). These PG receptors initiate multiple and distinct downstream signalling pathways (Figure 1) and thus function as double‐edged swords during diverse physiological and pathological processes where they can play conflicting roles (Narumiya & FitzGerald, 2001; Yao & Narumiya, 2019). In the healthy lung, NK and T cells express DP1 receptors while mast cells mildly express DP2 receptors. Among four PGE_2_ receptors, EP4 and EP2 receptors are expressed on stromal cells and nearly all immune cell types, but at very low levels on alveolar epithelial (AT1 or AT2) cells. IP receptors are highly expressed on stromal cells while TP receptors are expressed on vascular endothelial cells (Figure 2).

PGs are found in most mammalian cells and tissues and can promote both the initiation and resolution of inflammation, depending on local concentrations, disease setting, and timing of action (Yao & Narumiya, 2019). Fluctuations in expression of these synthases within cells recruited to sites of inflammation govern the production profiles of PGs. Although PGE_2_ is detectable in bronchoalveolar lavage (BAL) fluids (rather than bronchial wash fluids) taken from healthy individuals, its level (1.6 pM) is threefold to eightfold lower than levels of PGD_2_, PGF_2α_, and TXB_2_ (a stable TXA_2_ metabolite) (Gouveia‐Figueira et al., 2017). PG levels are usually elevated in response to inflammatory or noxious stimuli. For example, PGE_2_ compared to other PGs was found to be significantly up‐regulated in BAL fluids of individuals after exposure to biodiesel exhaust (Gouveia‐Figueira et al., 2017). Plasma levels of PGE_2_ were increased in patients from early stage (within the first week, 1,966 pg·ml^−1^ ≈ 5.6 nM) of SARS‐CoV‐1 infection and lasted to the late stage (2–3 weeks, 2,170 pg·ml^−1^ ≈ 6.2 nM) of infection compared to that in plasma from control individuals (1,300 pg·ml^−1^ ≈ 3.7 nM) (Lee et al., 2004). This is associated with induction of COX‐2 expression as the SARS‐CoV‐1 N protein binds directly to two regulatory elements (i.e., NF‐κB and C/EBP binding sites) of the COX‐2 promoter (Yan et al., 2006). By performing proteomic and metabolomic profiling of sera from COVID‐19 patients and healthy individuals, Shen et al. (2020) found a remarkable elevation of serum amyloid A1 (SAA1), SAA2, SAA4, and other inflammation markers (e.g., C‐reactive protein, SAP, and SERPINA3) in severe COVID‐19 patients compared to non‐severe COVID‐19 patients and healthy individuals. SAAs can enhance COX‐dependent AA metabolism and production of PGs, including PGE_2_, PGF_2α_, and TXA_2_, in many types of human cells, such as monocytes, macrophages, and fibroblasts, through NF‐κB activation (Li et al., 2017; Malle et al., 1997). Similarly, Yan et al. (2020) found up‐regulation of genes associated with the PGE_2_ synthesis pathway including PLAs, COX‐1, COX‐2, and PTGES3 during the disease progression of COVID‐19 patients, compared with that of healthy individuals in a longitudinal transcriptome analysis of peripheral blood mononuclear cells. In agreement with these findings, Hong et al. (2020) observed significantly higher (~10‐folds) levels of PGE_2_ in urine samples of COVID‐19 patients, compared with healthy individuals. PGE_2_ in COVID‐19 patient's urine remained at high levels for 5–12 days post‐hospital admission with routine treatment, but its level was reduced after taking oral celecoxib, a selective COX‐2 inhibitor. However, urinary PGE_2_ formed in the kidney does not represent systemic PG levels. To assess systemic production of PGs, detection of circulation levels of their metabolites, for example, PGE‐M, PGD‐M, and PGI‐M, in both blood and urine is needed. Moreover, PGs and their metabolites present in the lung, for example, in BAL fluid, will also be highly informative.

Traditionally, PGs have been viewed as inflammatory mediators connecting innate immunity to phases of acute inflammation with proinflammatory cytokines (TNF‐α and IL‐1β) and LPS known to induce expression of inducible COX‐2 and mPGES‐1 (Díaz‐Muñoz, Osma‐García, Cacheiro‐Llaguno, Fresno, & Iñiguez, 2010). However, COX‐2 and mPGES‐1 expression is also observed in chronically inflamed tissues including joints of rheumatoid arthritis patients, colons of patients with inflammatory bowel disease, and cancerous tumours and their micro‐environment (Ricciotti & FitzGerald, 2011; Wang & DuBois, 2018). Therefore, PGs appear to play integral roles during both acute inflammation and chronic inflammatory diseases. Gordon et al. systemically mapped the interaction landscape between SARS‐CoV2 proteins, including four structural (S, E, M, N) proteins and non‐structural proteins (Nsp), and human host cell proteins, and predicted numerous host proteins as potential drug targets. Remarkably, they found that non‐structural proteins Nsp7 and Nsp14 interacted with PTGES2 (encoding mPGES‐2) and PRKACA (encoding the catalytic subunit α of PKA), respectively (Gordon et al., 2020). mPGES‐2 mediates biosynthesis of PGE_2_, which mainly activates the cAMP–PKA pathway, and its receptors EP4 and EP2 are highly expressed in lung cells. Thus, targeting mPGES‐2 by NSAIDs (such as indomethacin, Yamada, Komoto, Watanabe, Ohmiya, & Takusagawa, 2005) can not only inhibit PGE_2_ production but also interrupt virus–host cell interaction. In addition, mPGES‐1 inhibition may be of clinical benefit during PGE_2_‐induced inflammatory episodes, including COVID‐19‐associated ARDS (Smeitink et al., 2020). Thus, targeting respective PG synthase might represent alternative and effective strategies in attempt to alleviate adverse side effects of NSAIDs that result from non‐specific inhibition of all PGs downstream of PGH_2_.

## 4 PGs IN COVID‐19: POSSIBLE IMMUNOPATHOLOGICAL MECHANISMS

What may be the underlying scientific rationale and biological mechanisms for either beneficial or harmful effects of NSAIDs on the risk of development of COVID‐19 or disease severity? What are the fundamental mechanisms underlying the risk factors (e.g., age, male sex, and underlying healthy conditions) for developing severe to critical disease and death in COVID‐19 patients (Docherty et al., 2020; Williamson et al., 2020)? Are PGs involved in the risks of development of severe COVID‐19 disease, and if yes, how do PGs fundamentally function at various stages of SARS‐CoV‐2 infection, virus–host interactions, and during pathogenesis of COVID‐19? Answers for such questions remain vague.

### 4.1 Potential roles of AA in COVID‐19

AA is known to have potent antimicrobial capacity including leakage and lysis of microbial cell membranes, viral envelope disruption, amino acid transportation, inhibition of respiration, and uncoupling of oxidative phosphorylation (Das, 2018). It is reasonable to suggest that a variety of immune cells, including neutrophils, alveolar macrophages, B and T lymphocytes, and NK cells, liberate AA and other unsaturated fatty acids to the immediate extracellular space when challenged by CoVs including SARS‐CoV‐1, MERS‐CoV, and possibly SARS‐CoV‐2 (Das, 2020). Indeed, Yan et al. (2019) found that HCoV‐229E infection markedly increased the levels of cPLA_2_‐dependent glycerophospholipids and fatty acids including Linoleic acid and AA and that supplementation with exogenous linolenic acid or AA inhibited virus replication of HCoV‐229E and MERS‐CoV in Huh‐7 cells. Accordingly, Shen et al. (2020) used metabolomic assays to show that serum levels of AA (20:4n6) were significantly reduced in COVID‐19 patients which was negatively associated with disease severity, suggesting that decreased levels of AA may lead to lack of inhibition of SARS‐CoV‐2 replication in COVID‐19 patients. In light of this, it is suggested that oral or intravenous administration of AA and other unsaturated fatty acids may enhance recovery in COVID‐19 patients (Das, 2020). On the other hand, decrease of AA levels could also be explained as a result of facilitation of AA metabolism, that is, conversion into PGs. Indeed, up‐regulated gene expression of COX‐1, COX‐2, and PTGES3 and increased PGE_2_ levels are found in COVID‐19 patients (Hong et al., 2020; Yan et al., 2020). Furthermore, cPLA_2_α serves to release of AA from cell membrane lipids and subsequent production of PGs. In the human lung, cPLA_2_ genes are highly expressed in endothelial and epithelial cells (Figure 2) where CoVs (SARS‐CoV‐1 and SARS‐CoV‐2) first attach. Expression of several PLA_2_ genes (e.g., PLA2G4A, PLA2G4C, PLA2G7, and PLA2G15) is up‐regulated in COVID‐19 patients compared to healthy controls, and their expression reduces to normal levels when patients recover from COVID‐19 (Yan et al., 2020). Inhibition of cPLA_2_α activity using pyrrolidine‐2 significantly reduced viral replication and formation of double membrane vesicles in HCoV‐229E‐infected Huh‐7 cells and MERS‐CoV‐infected Huh‐7/Vero cells (Müller et al., 2018), implying that anti‐CoV treatments harnessing cPLA_2_α inhibition may be of potential therapeutic benefit. It remains unknown whether cPLA_2_α and its products (i.e., fatty acids) regulate CoV replication directly or indirectly through their further downstream metabolites of fatty acids, for example, PGs. Below, we will focus on discussion of possible functions of PGD_2_ and PGE_2_ even though other PGs may also influence SARS‐CoV‐2 infection and COVID‐19. For example, PGI_2_ and TXA_2_ respectively can reduce and promote thrombosis, a typical complication that occurs in nearly half of critically ill COVID‐19 patients and which contributes to mortality (Klok et al., 2020; Wise, 2020). Moreover, the vasodilatory and endothelial cell effects of PGs are well known. However, here, we will focus on discussing the roles of PGs in COVID‐19 immunopathology.

### 4.2 Potential roles of PGs on thrombosis in COVID‐19

The exact mechanisms behind the development of systemic coagulopathy and acquired thrombophilia defined in the majority of COVID‐19 cases which can lead to venous, arterial, and microvascular thrombosis remain unclear (Becker, 2020). Indeed, clinical characteristics of COVID‐19 include elevated D‐dimer levels, prolonged thrombin time, and thrombocytopenia (platelet count <150,000·μl^−1^), therefore suggesting an increased possibility of disseminated intravascular coagulation or pre‐disseminated intravascular coagulation (Guan et al., 2020). Moreover, pooled analysis suggests that significant increases in D‐dimer levels as a predictor of adverse outcomes were regularly observed in COVID‐19 patient blood, implying the presence of underlying coagulopathy (Lippi & Favaloro, 2020). Although D‐dimer levels can be altered by numerous inflammatory processes, in cases of COVID‐19, it is almost certainly due to intravascular thrombosis (Cui, Chen, Li, Liu, & Wang, 2020; Leonard‐Lorant et al., 2020). A retrospective cohort study found that on admission, increased D‐dimer levels (>1,000 ng·ml^−1^) were associated with increased risk of death in hospitalised COVID‐19 patients (F. Zhou, Yu, et al., 2020). In COVID‐19 patients, increased D‐dimer levels continue to be a persistent marker of poor outcome (Bikdeli et al., 2020). Klok et al. (2020) reported a high incidence of 31% thrombotic complications in ICU patients with COVID‐19, of which venous thromboembolic events were most common (27%). Furthermore, in 183 consecutive patients with COVID‐19 pneumonia, abnormal coagulation parameters and poor prognosis were recorded, and patients who died (compared to survivors) exhibited increased D‐dimer levels, fibrinogen degradation products, and longer PT and APTT values (Tang, Li, Wang, & Sun, 2020). Pathological characteristics of COVID‐19 infection include platelet‐fibrin thrombosis and intravascular megakaryocytes in all major organs, including the heart and lungs (Fox et al., 2020). Megakaryocytes are responsible for the production of platelets; therefore, the employment of anti‐platelet agents may be of clinical benefit during COVID‐19 pathogenesis. Aspirin is a broadly studied anti‐platelet drug which exerts its cardioprotective effects via irreversible inhibition of platelet COX‐1, thus blocking TXA_2_ production in activated platelets, and so decreases prothrombotic events. However, aspirin does not confer platelet‐specific effects, and in other cell types (via inhibition of COX‐1 and in some cases COX‐2), it can decrease prostanoid production, for example, PGI_2_, which serves to inhibit platelet aggregation (conversely to TXA_2_). Interestingly, as determined by a pharmacodynamic interaction study in healthy volunteers, other NSAIDs including ibuprofen, naproxen, indomethacin, and tiaprofenic acid all block the anti‐platelet effect of aspirin, whereas celecoxib and sulindac did not exhibit any significant anti‐platelet effects (Gladding et al., 2008). Although aspirin can inhibit viral replication and confer anti‐inflammatory and anti‐coagulant effects, at present, it has not been thoroughly investigated in the treatment of thrombosis during COVID‐19. However, COVID‐19 clinical trials involving aspirin administration are ongoing. That said, PGs (whose production is blocked by NSAIDs) can also confer anti‐platelet effects and therefore also merit clinical attention in the context of COVID‐19. For example, it has been long understood that within the PG family, PGE_1_ is the most potent inhibitor of ADP‐induced platelet aggregation whereas PGE_2_ possesses roughly a fifth of its activity (Irion & Blombäck, 1969). PGE_2_–EP4 receptor signalling does effectively inhibit platelet aggregation at high concentrations (>1 × 10^−6^ M) (Macintyre & Gordon, 1975), and akin to PGI_2_, PGD_2_ can also inhibit platelet aggregation (Smith, Silver, Ingerman, & Kocsis, 1974), but PGE_2_–EP3 receptor signalling augments platelet aggregation (Friedman, Ogletree, Haddad, & Boutaud, 2015).

### 4.3 Potential roles of PGD_2_ in COVID‐19

Ageing is associated with up‐regulated gene expression of PG synthases (e.g., COX‐2 and mPGES‐1) and elevated levels of PGs (e.g., PGE_2_, PGD_2_, PGF_2α_, and TXB_2_) in humans (Li et al., 2015). Similarly, Vijay et al. (2015) found that expression of PLA_2_ group IID (PLA2G2D) was increased in aged mouse lungs compared to younger mice, leading to augmented production of PGD_2_, PGE_2_, PGF_2α_, and TXB_2_ in lungs in response to SARS‐CoV‐1 infection. Differential gene expression showed that the major source of PLA2G2D was lung‐resident CD11c^+^ cells, for example, alveolar macrophages and DCs (Vijay et al., 2015). Strikingly, aged mice with deficiency in PLA2G2D were protected from SARS‐CoV‐1 infection, exhibited enhanced virus‐specific cytotoxic CD8 T cell responses, and increased migration of respiratory DCs to draining lymph nodes (Vijay et al., 2015). PGD_2_ may contribute to SARS‐CoV‐1 infection, as treatment with exogenous PGD_2_ in young mice decreased respiratory DC migration, while treatment with a DP1 receptor antagonist enhanced respiratory DC migration, CD8 T cell responses, and the kinetics of virus clearance in lungs of aged, SARS‐CoV‐1‐infected mice (Zhao, Zhao, Legge, & Perlman, 2011). Enhanced T cell responses are of integral importance because reduced T cell responses are associated with higher mortality rates in SARS‐CoV‐1‐infected mice (Channappanavar, Zhao, & Perlman, 2014; Zhao, Zhao, Van Rooijen, & Perlman, 2009) and lymphopenia is also associated with disease progression of COVID‐19 (Tan et al., 2020). Vijay et al. (2017) further showed that PGD_2_–DP1 receptor signalling works together with type I IFN signalling to induce expression of the pyrin domain only‐containing protein 3 (PYDC3) that diminishes neurotropic CoV‐induced inflammasome activation and proinflammatory cytokine (such as IL‐1β) expression in mouse brain, thus preventing chronic inflammation and tissue damage. Interestingly, DP1 receptor signalling in human macrophages also up‐regulates POP3, a putative functional analogue of mouse PYDC3, suggesting that PGD_2_ similarly modulates inflammasomes in human cells (Vijay et al., 2017). These studies thus suggest that targeting the PGD_2_– DP1 receptor pathway may be helpful to control SARS‐CoV infection in older humans. Furthermore, SARS‐CoV‐2 may cause mast cell activation via the toll‐like receptors (TLRs) or inducing crosslink of IgE–FcεRI and subsequent production of PGD_2_, where use of mast cell stabilisers, including β‐adrenoceptor agonists, is highlighted as potential therapeutic targets during SARS‐CoV‐2 infection due to their efficient inhibition of PGD_2_ release (Kilinç & Kilinç, 2020). Infection with respiratory syncytial virus (RSV) up‐regulates HPGDS expression and increases PGD_2_ secretion by cultured human primary airway epithelial cells (Werder et al., 2018). Blocking the DP2 receptor decreased viral load, immunopathology, and morbidity in a neonatal mouse model of severe viral bronchiolitis (Werder et al., 2018). Interestingly, the beneficial effects of DP2 receptor antagonism are inhibited by concurrent blocking of the DP1 receptor, and DP1 receptor agonists up‐regulate type III IFN (i.e., IFN‐γ) production and IFN‐stimulated gene (ISG) expression, accelerating viral clearance (Werder et al., 2018). This finding suggests reciprocal effects of DP1 and DP2 receptors in RVS infection, although it is contrary to the findings of the pathogenic role of DP1 receptors during SARS‐CoV‐1 infection (Zhao et al., 2011). Therefore, investigations should attempt to analyse the actions of PGD_2_, DP1 receptors and DP2 receptors on SARS‐CoV‐2 infection and during development of COVID‐19.

### 4.4 Potential roles of PGE_2_ in COVID‐19

**FIGURE 3:**
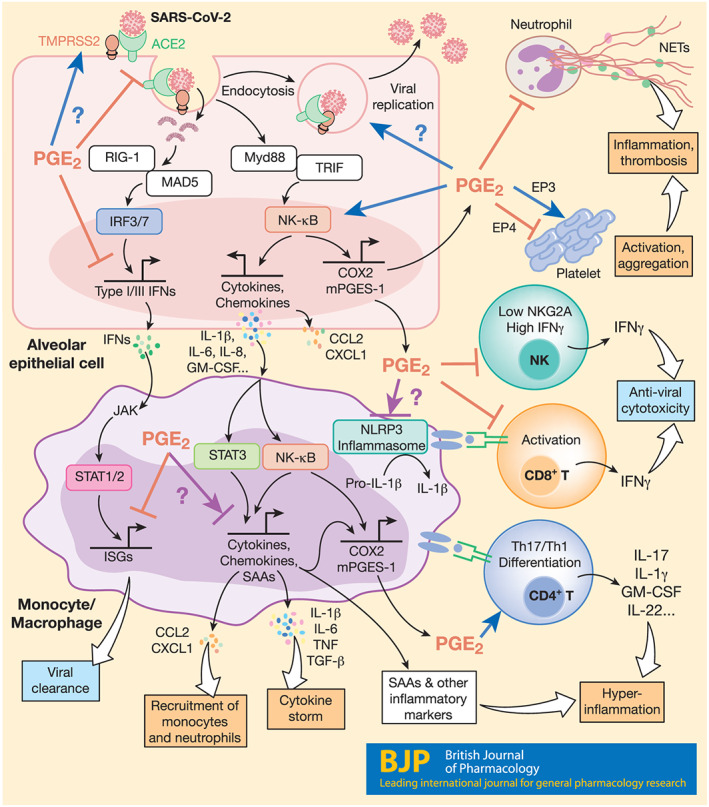
Possible mechanisms for PGE_2_ modulation of immune cell functions in COVID‐19. PGE_2_ is likely to modulate immune responses in various cell types during SARS‐CoV‐2 infection, influencing COVID‐19 pathogenesis. In epithelial cells, attachment of SARS‐CoV‐2 with ACE2 and TMPRSS2 leads to endocytosis, viral replication, and cell damage, activating RLR (RIG‐1 and MAD5)‐dependent production of type I and III IFNs and the TLR‐dependent NF‐κB pathway. The NF‐κB pathway induces expression of proinflammatory cytokines (e.g., IL‐1b, IL‐6, IL‐8, and GM‐CSF), chemokines (e.g., CCL2 and CXCL1), and other inflammatory mediators such as COX‐2 and mPGES‐1, resulting in PGE_2_ secretion. Here, while it suppresses production of type I (and possibly type III) IFNs, PGE_2_ further amplifies NF‐κB signalling and production of cytokines and chemokines in a positive feedback loop. PGE_2_ may also directly modulate ACE2 and TMPRSS2 gene expression, endocytosis, and viral replication. In monocytes/macrophages, activation of NF‐κB and STAT3 mediates production of large amounts of inflammatory cytokines which contributes to development of cytokine secretion syndrome (the “cytokine storm”), chemokines that recruit monocytes and neutrophils, inflammatory biomarkers (e.g., SAAs, CPR, and D‐dimer), and PGE_2_. Here, PGE_2_ again represses IFN‐induced expression of ISGs, contributing to delay of viral clearance. Importantly, PGE_2_ context‐dependently affects (either positively or negatively) not only NLRP3 inflammasome activation and related IL‐1β maturation but also NF‐κB‐dependent monocyte/macrophage cytokine production. PGE_2_ differentially regulates platelet aggregation via different receptors and probably inhibits NETosis associated with inflammation and thrombosis. PGE_2_ also down‐regulates IFN‐γ production and cytotoxicity of NK and CD8 T cells that kill cells infected with SARS‐CoV‐2 but promotes differentiation of proinflammatory Th17 and Th1 cells, the chief cellular sources of the cytokine storm at late stages of COVID‐19

PGE_2_ is considered an anti‐inflammatory agent in various lung conditions including ALI, asthma, fibrosis, and bacterial infections (Birrell et al., 2015; Felton et al., 2018; Lundequist et al., 2010; Vancheri, Mastruzzo, Sortino, & Crimi, 2004). But it can also exert inflammatory effects in certain condition such as COPD, lung cancer, and certain viral infections (Bonanno et al., 2016; Dehle et al., 2013; Nakanishi & Rosenberg, 2013). Infection with a wide range of viruses, for example, herpes simplex virus, rotavirus, and influenza A virus (IAV), induces expression of COX‐2 and mPGES‐1, resulting in overproduction of PGE_2_, which in turn plays a role in viral infection directly by modulating viral binding, replication, and gene expression and/or indirectly by regulating the host immune responses (see Sander, O'Neill, & Pohl, 2017). Indeed, elevated levels of PGE_2_ were observed in patients infected with SARS‐CoV‐1 or SARS‐CoV‐2 (Hong et al., 2020; Lee et al., 2004). Furthermore, Smeitink et al. (2020) speculate that in males, increased PGE_2_ may correlate with enhanced COVID‐19 severity by augmenting thrombotic responses. This may be in part due to effects of PGE_2_ in promoting intravascular thrombosis via EP3 receptors in platelets (Gross, Tilly, Hentsch, Vonesch, & Fabre, 2007). Elevated levels of PGE_2_ may further enhance SARS‐CoV‐2 cell entry. Indeed, elevated PGE_2_ does enhance clathrin‐mediated endocytosis of bovine ephemeral fever virus (Cheng, Huang, Chi, Chiu, & Liu, 2015). Here, we will discuss potential actions of PGE_2_ in COVID‐19 based on the current state of the knowledge of this lipid in modulation of immune responses during infections or under inflammatory conditions (Figure 3).

#### 4.4.1 PGE_2_ on IFN signalling

Infection with SARS‐CoV‐2 ligates various pathogen recognition receptors, for example, TLRs and/or RIG‐I‐like receptors, and activates transcription factors such as IFN regulatory factor 3 (IRF3) and NF‐κB that are responsible for expression of type I and III IFNs and proinflammatory mediators, including TNF‐α, IL‐6, and PGE_2_ respectively. Secreted IFNs then activate the JAK‐STAT1/2 pathway to trigger production of ISGs that directly recognise and execute antiviral functions (Park & Iwasaki, 2020). PGE_2_ has been reported to inhibit production of type I IFNs. For example, after infection with IAV, mPGES‐1 deficient macrophages exhibit an early increase in phospho‐IRF3, compared to wild‐type (WT) macrophages, augmented type I IFN, and decreased viral load, which was drastically inhibited by addition of exogenous PGE_2_ (Coulombe et al., 2014). Fabricius et al. (2010) reported that PGE_2_ inhibited secretion of IFN‐α by TLR‐activated human plasmacytoid DCs through its EP2 and EP4 receptor‐mediated suppression of IRF7. Patients with mild to moderate COVID‐19 had high type I IFN responses, while severe COVID‐19 patients had impaired type I IFN activity and down‐regulated ISGs (Hadjadj et al., 2020). It remains to be clarified whether severe COVID‐19 patients have higher levels of PGE_2_, and if so, whether this represents a mechanism evolved by SARS‐CoV‐2 for suppression of type I IFN production and function. Moreover, as DP1 receptors promote type III IFN production and signalling and contributes to RSV viral clearance (Werder et al., 2018) and PGE_2_ receptors (EP2 and EP4) both activate the cAMP–PKA pathway (as do DP1 receptors), it would be of interest to investigate whether PGE_2_ has different effects on production and functions of type I and III IFNs during SARS‐CoV‐2 infection.

#### 4.4.2 PGE_2_ on NF‐κB signalling

Viral replication triggers hyperinflammation and cytokine storm syndrome, a leading cause of mortality in severe COVID‐19 patients (Mehta et al., 2020). Proinflammatory cytokines such as IL‐1, IL‐6, IL‐8, and TNF‐α and chemokines including CCL2, CCL3 and CXCL10 are produced from epithelial cells, monocytes, and macrophages, shortly after clinical symptoms appear (Mehta et al., 2020). Many clinical trials have been set up to treat COVID‐19 by targeting pathways relating to proinflammatory cytokine production, for example, IL‐1 and IL‐6 (Bonam, Kaveri, Sakuntabhai, Gilardin, & Bayry, 2020; Merad & Martin, 2020). NF‐κB is the key transcription factor responsible for induction of proinflammatory cytokines. Activation of NF‐κB can stimulate gene expression of inducible COX‐2 and mPGES‐1 in many cell types, leading to production of COX‐2‐dependent PGE_2_. This PGE_2_ acts autocrinally and/or paracrinally on NF‐κB stimulation for expanding of proinflammatory cytokines and chemokines through the EP2 (maybe also EP4) receptors (Aoki et al., 2017; Yao & Narumiya, 2019). For example, PGE_2_ mediates NF‐κB‐dependent IL‐8 gene transcription and protein secretion in human cells such as pulmonary microvascular endothelial cells, nasal polyp‐derived fibroblasts, and HEK293 cells, most likely through the EP4 receptor (Aso et al., 2012; Cho, Han, Lee, & Lee, 2014; Neuschäfer‐Rube, Pathe‐Neuschäfer‐Rube, Hippenstiel, Kracht, & Püschel, 2013). Thus, amplification of the PGE_2_–NK‐κB positive feedback system is likely to contribute to the cytokine storm and hyperinflammation observed in COVID‐19.

#### 4.4.3 PGE_2_ on IL‐6 production

Elevated IL‐6 and IL‐6‐activated genes have been found in peripheral blood of COVID‐19 patients (Hadjadj et al., 2020; Huang et al., 2020). PGE_2_ has been reported to have contradictory effects on IL‐6 production which probably depends on the stimuli. For example, PGE_2_ acting on EP4 receptors inhibits LPS‐induced proinflammatory cytokines (e.g., IL‐6 and TNF‐α) and promotes systemic inflammation in multiple organs of mice (Duffin et al., 2016). Furthermore, PGE_2_– EP4 receptor signalling was also shown to suppress LPS‐induced ALI partially through IL‐6 and TNF‐α production (Birrell et al., 2015; Felton et al., 2018). PGE_2_ also inhibited LPS‐ and *Streptococcus pneumoniae*‐induced IL‐6 and TNF‐α production from human lung macrophages (Gill et al., 2016). Conversely, however, injection of mineral oils induced peritoneal macrophages to release IL‐6 and PGE_2_, which was reversed by inhibition of endogenous PGE_2_ signalling (Hinson, Williams, & Shacter, 1996). In humans, PGE_2_ promotes IL‐1β‐dependent production of IL‐6, M‐CSF, and VEGF from human fibroblasts via EP4 receptors (Inoue et al., 2002). PGE_2_ also enhances induction of IL‐6 and other proinflammatory cytokines (e.g., IL‐8) upon various stimuli in monocytes, macrophages, fibroblasts, and airway epithelial cells through both EP2 and EP4 receptors (B.‐C. Chen et al., 2006; Cho et al., 2014; Li, Qi, & Cowley, 2011; Raychaudhuri, Douglas, & Smith, 2010). In return, IL‐6 further up‐regulates COX‐2 gene expression and increases PGE_2_ production, working together for optimised production of other inflammatory factors, for example, MMP9 (Kothari et al., 2014). Moreover, PGE_2_ can promote IL‐6 production in a paracrine way. For example, PGE_2_ promotes Th17 cells to secrete IL‐17A which then stimulates fibroblast to produce proinflammatory cytokines, for example, IL‐6, IL‐8, and IL‐1β (Paulissen et al., 2013).

#### 4.4.4 PGE_2_ on inflammasome activation

Inflammasomes play a critical role in the formation of cytokine storms commonly observed in SARS patients and possibly have similar functions in COVID‐19 patients. SARS‐CoV‐1 E protein activates not only NF‐κB but also the inflammasome NLRP3 (NOD‐, LRR‐, and pyrin domain‐containing protein 3), leading to secretion of IL‐1β and IL‐18 (Nieto‐Torres et al., 2015). SARS‐CoV‐1 open reading frame (ORF) 3a and ORF8b can also activate NLRP3 inflammasome (C.‐S. Shi, Nabar, Huang, & Kehrl, 2019; Siu et al., 2019). It was reported that SARS‐CoV‐2 S protein primes NLRP3 inflammasome activation and IL‐1β secretion in macrophages derived from COVID‐19 patients but not in macrophages from healthy SARS‐CoV‐2 naïve controls and that chemical inhibition of NLRP3 blocks spike protein‐induced IL‐1β secretion ex vivo (Theobald et al., 2020). PGE_2_ promotes pro‐IL‐1β gene expression and IL‐1β secretion via a positive feedback loop (Zasłona et al., 2017). In a mouse model of *Pseudomonas aeruginosa* bacterial infection, PGE_2_ mediates IL‐1β induction, limits autophagy‐mediated killing of *P. aeruginosa* in alveolar macrophages, and augments IL‐1β‐mediated ALI (Martínez‐Colón, Taylor, Wilke, Podsiad, & Moore, 2018). But there are contradictory findings on how PGE_2_ modulates NLRP3 inflammasome activation. PGE_2_ was reported to inhibit NLRP3 inflammasome activation in macrophages through EP4 receptors, and blockade of COX‐2 or EP4 receptors resulted in increased NLRP3 inflammasome activation (Mortimer, Moreau, MacDonald, & Chadee, 2016; Sokolowska et al., 2015). However, other authors report positive effects of PGE_2_ upon NLRP3 inflammasome activation and IL‐1β production in macrophages and monocytes under various stimuli (Nakata et al., 2020; Sheppe et al., 2018; Zasłona et al., 2017). Taken together, these findings indicate context‐dependent modulation of NLRP3 inflammasome activation by PGE_2_. Careful examinations are thus required to clarify the effects of PGE_2_ upon NLRP3 inflammasome activation, for example, primed by different SARS‐CoV‐2 proteins in different cell types.

#### 4.4.5 PGE_2_ on monocyte/macrophage functions

Monocytes and macrophages are main sources of proinflammatory and anti‐inflammatory cytokines and generally function to eliminate pathogens. Expansion of IL‐6‐producing CD14^+^CD16^+^ monocytes was observed in peripheral blood from severe COVID‐19 patients (X. Zhang, Tan, et al., 2020; Y. Zhou, Fu, et al., 2020), but reduction of HLA‐DR on CD14^+^ monocytes was found in COVID‐19 patients with severe respiratory failure, which was associated with increased IL‐6 (Giamarellos‐Bourboulis et al., 2020), indicative of immunosuppression. This scenario is likely to involve the immuno‐suppressant PGE_2_ that can be secreted by both human monocytes and macrophages. While human macrophages use an IL‐1β‐independent pathway exclusively, monocytes produce PGE_2_ by both IL‐1β‐independent and IL‐1β‐dependent pathways; the latter involving TLR4/TRIF/IRF3 signalling (Endo, Blinova, Romantseva, Golding, & Zaitseva, 2014). Qiu et al. (2017) found an increase of CD14^+^CD16^+^ monocytes in patients with severe sepsis or septic shock, which was positively associated with disease severity. After in vitro culture of monocytes from sepsis patients, PGE_2_ diminished CD14^+^CD16^+^ monocytes after 24 h, reduced TNF‐α production, but enhanced anti‐inflammatory IL‐10 production (Qiu et al., 2017). High amounts of IL‐1β and PGE_2_ are mainly produced from the classic inflammatory CD14^+^CD16^−^ human monocytes after *Candida albicans* infection (Smeekens et al., 2011). Increase in inflammatory monocytes was indicated to be associated with increased survival rate at least in Gram‐negative sepsis patients (Gainaru et al., 2018). Hence, reduction of inflammatory CD14^+^ monocytes by PGE_2_ may sustain immunosuppression and could be associated with poor clinical outcome. Given the important roles of monocytes and their abilities to differentiate into macrophages and DCs, it is imperative to examine the effects of PGs on functions of each subpopulations of monocytes under different stimulatory conditions of SAR‐CoV‐2 infection.

Single‐cell RNA sequencing analysis of cells in BAL fluid from COVID‐19 patients suggested that monocyte‐derived macrophages, but not alveolar macrophages, contribute to lung inflammation and damage in severe COVID‐19 patients (Liao et al., 2020). Bulk RNA sequencing analysis of BAL cells also suggested increased production of chemokines such as CCL2 and CXCL1 (Liao et al., 2020; Xiong et al., 2020; Z. Zhou, Ren, et al., 2020), which recruit CCR2‐expressing classical monocytes and neutrophils, respectively, to the lung from peripheral blood. Interestingly, PGE_2_–EP2 receptor signalling increases CCL2 and CXCL1 production from macrophages and other cells (Aoki et al., 2017; Yao & Narumiya, 2019). During human monocyte/macrophage differentiation, cAMP‐elevating reagents such as PGE_2_ can cause a large increase in the mRNA and protein levels of several proinflammatory CCL and CXCL chemokines, contributing to the pathogenesis of lung disease (Hertz et al., 2009). Furthermore, alternatively activated M2 macrophages differentiated from monocytes promote tissue repair by secreting reparative cytokines such as TGF‐β, amphiregulin (AREG) and VEGF (Wynn & Vannella, 2016). As PGE_2_ facilitates generation of M2 macrophages, it may thus also contribute to lung fibrosis in severe COVID‐19 patients.

#### 4.4.6 PGE_2_ on NET release

The formation of neutrophil extracellular traps (NETs) is an evolutionarily ancient process which involves the release of de‐condensed nuclear chromatin studded with various antimicrobial proteins, such as core histones, neutrophil elastase and MPO, to the extracellular space where they serve to trap and kill invading microorganisms (Brinkmann et al., 2004; Fuchs et al., 2007; Robb, Dyrynda, Gray, Rossi, & Smith, 2014). However, aberrant NET formation is involved in a wide range of NET‐associated diseases. Hence, NETs are regarded as double‐edged swords in innate immunity (Kaplan & Radic, 2012). Given the important role played by NETs in the pathogenesis of various respiratory diseases and thrombosis, many researchers also suggest NETs as key players in the pathogenesis of COVID‐19, most probably via NET‐mediated release of excessive amounts of IL‐6 and IL‐1β during cytokine storms in the COVID‐19 milieu (Barnes et al., 2020; Mozzini & Girelli, 2020; Thierry, 2020; Thierry & Roch, 2020; Tomar, Anders, Desai, & Mulay, 2020). Indeed, there is a high degree of NET–IL‐1β interplay during both venous and arterial thrombosis and severe asthma (Lachowicz‐Scroggins et al., 2019; Liberale et al., 2019; Yadav et al., 2019), and it has been hypothesised that there may be therapeutic potential in targeting the IL‐1β/NET feedback loop (Yaqinuddin & Kashir, 2020). It is proposed that upon SARS‐CoV‐2 infection, activated endothelial cells recruit neutrophils where they release NETs, which in turn activates the contact pathway of coagulation, subsequently trapping and activating platelets to potentiate blood clotting (Merad & Martin, 2020). NETs contribute to immunothrombosis in COVID‐19 ARDS where pulmonary autopsies confirmed NET‐associated microthrombi with neutrophil–platelet infiltration (Middleton et al., 2020). Such NET‐induced immunothrombosis may help explain the prothrombotic clinical presentations observed in COVID‐19 patients (Middleton et al., 2020). Indeed, NETs have been identified as potential markers of disease severity in COVID‐19 (Zuo et al., 2020), and elevated NET formation in hospitalised COVID‐19 patients is associated with higher risk of thrombotic episodes (Zuo et al., 2020). Serum samples of hospitalised COVID‐19 patients contained greater cell‐free DNA and hallmark NET‐associated products, including MPO–DNA complexes and citrullinated histone H3, compared to healthy control serum samples (Zuo et al., 2020). Furthermore, serum of COVID‐19 patients requiring mechanical ventilation exhibited augmented cell‐free DNA and MPO–DNA complexes, compared to patients breathing room air (Zuo et al., 2020). An additional NET marker, calprotectin, was found to be present at prominently elevated levels in the blood of 172 COVID‐19 patients (H. Shi, Zuo, et al., 2020). PGs are reported to have inhibitory effect upon NET formation. PGE_2_ inhibited NET formation after stem cell transplant (Domingo‐Gonzalez et al., 2016) and via EP2‐ and EP4 receptor‐mediated activation of cAMP (Shishikura et al., 2016). After co‐culture of neutrophils with cAMP‐elevating reagents, PMA‐induced NET formation was significantly reduced (Shishikura et al., 2016). The adenylate cyclase toxin which vastly increases intracellular cAMP is also known to reduce NETs (Eby, Gray, & Hewlett, 2014). Interestingly, induction of intracellular cAMP production by PGE_1_ markedly constrains NETs induced by the pancreatic cancer cell line AsPC‐1 (Jung et al., 2019). Importantly, CGS21680, a selective agonist of the adenosine A_2A_ receptor (which increases intracellular cAMP), successfully diminished NET formation mediated by antiphospholipid antibodies, which increased the incidence of thrombotic events (Ali et al., 2019). Furthermore, in mice treated with antiphospholipid antibodies, CGS21680 impaired thrombosis within the inferior vena cava (Ali et al., 2019). Here, the authors also demonstrate similar inhibition of NETs via dipyridamole, an antithrombotic medication which increases extracellular adenosine and impedes cAMP breakdown (Ali et al., 2019). Together with the known inhibitory effects of PGE_2_ on platelet function (Gross et al., 2007), such evidence suggests that there may be the potential for therapeutic gain in harnessing existing drugs that augment production of PGs and cAMP and thus reduce excess NET formation and the incidence of thrombotic events during SARS‐CoV‐2 infection.

#### 4.4.7 PGE_2_ on T cell functions

T cells may play critical roles in SARS‐CoV‐2 infection and COVID‐19 pathogenesis. First, CD8^+^ cytotoxic T cells attack infected cells to prevent viral amplification. Second, CD4^+^ helper T cells help B cells produce antibodies and modulate innate immune responses. Third, hyperactivated and differentiated type 1 and 17 helper T cells (i.e., Th1 and Th17 cells, respectively) produce large amounts of proinflammatory cytokines (e.g., IL‐2, TNF‐α, IFN‐γ, IL‐6, and IL‐17) that contribute to the cytokine storm and immunopathology, but viral infection may also activate regulatory T (Treg) cells which limit immunopathology through mechanisms such as the production of anti‐inflammatory cytokines (e.g., IL‐10). After infection with SARS‐CoV‐2, a significant reduction of peripheral blood CD4^+^ and CD8^+^ T cells (a condition known as lymphopenia) results in moderate to severe COVID‐19 patients, which is correlated with disease severity and mortality (G. Chen, Wu, et al., 2020; Diao et al., 2020; Liu, Li, et al., 2020; Tan et al., 2020). In contrast to peripheral blood, mass lymphocyte infiltrations were observed in the lung as confirmed by post‐mortem examination of a COVID‐19 patient who suffered from ARDS (Tian et al., 2020). PGE_2_ has multifaceted effects on modulation of T cell responses (Yao & Narumiya, 2019). PGE_2_ suppresses T cell receptor‐dependent T cell activation and proliferation via EP2/EP4 receptor‐mediated cAMP–PKA pathway, but this suppressive effect is weakened by enhancing CD28 co‐stimulation through augmentation of PI3K signalling (Yao et al., 2013). Following SARS‐CoV‐2 infection, the pathway related to CD28 signalling in T helper cells was significantly down‐regulated, while the PKA pathway and PGE_2_ biosynthesis pathway were significantly up‐regulated (Z. Zhou, Ren, et al., 2020; Yan et al., 2020). Thus, PGE_2_–cAMP–PKA signalling is likely to inhibit antigen‐dependent activation of antiviral T cell responses in COVID‐19 patients. On this basis, use of NSAIDs by inhibiting endogenous PGE_2_ production may enhance antiviral T cell responses in COVID‐19 patients. Indeed, enhanced viral antigen‐specific CD8^+^ and CD4^+^ T cell responses in lungs were found in PTGES‐deficient mice where PGE_2_ production was largely reduced, compared to WT mice, post‐IAV infection, and this was associated with reduced viral load in PTGES‐deficient animals (Coulombe et al., 2014). Moreover, increased expression of the immune checkpoint proteins, PD‐1 (CD279) and TIM‐3 (CD366), on CD8^+^ T cells was detected in severe and critical COVID‐19 patients (Diao et al., 2020; Y. Zhou, Fu, et al., 2020), suggesting that T cell exhaustion may occur. Interestingly, PGE_2_ through EP2 and EP4 receptors synergies with PD‐1 signalling to suppress mice and human cytotoxic T cell survival and function during chronic viral infection or in the tumour micro‐environment. Combined blockade of COX–PGE_2_ signalling and PD‐1/PD‐L1 augments T cell responses and improves control of viral infection and tumour growth (J. H. Chen et al., 2015; Miao et al., 2017; Sajiki et al., 2019).

As for inflammatory T cells, PGE_2_ regulates Th1 cell differentiation dependently on the strengths of T cell receptor and CD28 co‐stimulation and timing of PGE_2_ encounter (Yao et al., 2013). Given the down‐regulation of CD28 signalling, immediate up‐regulation of gene‐related PGE_2_ synthases (i.e., *PTGS1*, *PTGS2*, and *PTGES3*) at the early stages post‐SARS‐CoV‐2 infection, and the kinetics of PGE_2_ secretion in COVID‐19 patients (Hong et al., 2020; Yan et al., 2020; Z. Zhou, Ren, et al., 2020), it is proposed that PGE_2_ may inhibit the development of inflammatory IFN‐γ‐producing Th1 cells, although further investigations are required. This possibility is further supported by the findings that PGE_2_ inhibits monocyte‐derived DCs and macrophages to produce IL‐12 (Kaliński, Hilkens, Snijders, Snijdewint, & Kapsenberg, 1997; van der Pouw Kraan, Boeije, Smeenk, Wijdenes, & Aarden, 1995), the key cytokine for generating IFN‐γ‐producing Th1 cells. Of note, IFN‐γ production from CD8^+^ T cells and NK cells was not significantly different in blood of non‐severe and severe COVID‐19 patients, but IFN‐γ^+^ CD4^+^ T cells were likely to be reduced in severe, compared to non‐severe, COVID‐19 patients (G. Chen, Wu, et al., 2020; Qin et al., 2020). Th17 cells highly express IL‐17A, IL‐17F, IL‐22, and GM‐CSF that contribute to COVID‐19 immunopathology. PGE_2_ stimulates IL‐17 and aryl hydrocarbon receptor signalling pathways in human and mouse T cells through EP2/EP4 receptor –cAMP–PKA signalling, necessary for generation of pathogenic Th17 cells (Boniface et al., 2009; Lee et al., 2019; Robb et al., 2018; Yao et al., 2009). Given that SARS‐CoV‐2 infection specifically up‐regulates IL‐17F signalling and aryl hydrocarbon receptor signalling (Z. Zhou, Ren, et al., 2020), PGE_2_ is assumed to promote inflammatory Th17 cell expansion and function in COVID‐19.

There are mixed findings regarding Treg cells in COVID‐19. Reduction of Treg cell frequencies was observed in severe COVID‐19 patients compared to non‐severe patients (G. Chen, Wu, et al., 2020; Qin et al., 2020), but Y. Shi, Tan, et al. (2020) reported an increase in Treg cell numbers in peripheral blood of mild COVID‐19 patients compared to the control group, and there was no difference in Treg cell numbers between mild/moderate and severe patients. Both stimulatory and inhibitory effects of PGE_2_ on human Treg cell generation and suppressive function were observed (Li et al., 2019; Schiavon et al., 2019). The definitive functions of PGE_2_ on Treg cells in peripheral blood and lungs of COVID‐19 patients remain to be determined.

#### 4.4.8 PGE_2_ on NK cell functions

Like T cells, NK cells are also depleted in peripheral blood of severe, but not mild, COVID‐19 patients (Wen et al., 2020; Wilk et al., 2020; Zheng et al., 2020). NK cell activation may also be impaired in COVID‐19 patients due to down‐regulation of CD107a and cytokines such as IFN‐γ and TNF‐α but increased expression of NKG2A/CD94 that inhibits NK cell cytotoxicity (Zheng et al., 2020). These studies suggest that NK cell numbers and function are impaired in severe COVID‐19 patients. PGE_2_ was found to inhibit not only NK cell production of IFN‐γ but also myeloid cell production of IL‐12 that is required for IFN‐γ production through EP4 receptors (Van Elssen et al., 2011). The PGE_2_– EP4 receptor–cAMP signalling pathway also suppresses the cytolytic activity of NK and CD8^+^ T cells by increasing expression of NKG2A/CD94 (Holt, Ma, Kundu, & Fulton, 2011; Park et al., 2018; Zeddou et al., 2005). Moreover, PGE_2_ inhibits CXCR3 ligands such as CXCL9 and CXCL10 from antigen‐presenting cells, preventing NK cell migration (Gustafsson et al., 2011). It is thus likely that PGE_2_ both directly and indirectly inhibits NK cell migration to the lung, their activation and cytotoxic function, in the context of COVID‐19. However, this remains to be confirmed.

### 4.5 Potential roles of PGI_2_ in COVID‐19

Most human lung cells including stromal (fibroblasts), immune (monocytes, macrophages, and lymphocytes), and vascular endothelial cells express PGI_2_ synthase (PGIS) and the PGI_2_ receptor IP (Figure 2). As PGI_2_ also activates the cAMP–PKA signalling pathway, as does PGE_2_–EP2/EP4 receptor signalling, it is assumed that these two molecules may share similar effects on modulation of immune and inflammatory responses (see Dorris & Peebles, 2012) although PGI_2_ has been less extensively studied, compared with PGE_2_. Firstly, PGI_2_ is likely to suppress virus (e.g., RSV)‐induced type I IFN production in the lung, which contributes to protection against viral infection (Hashimoto et al., 2004; Toki et al., 2013). Overexpression of PGIS in bronchial epithelium decreased viral replication and limited weight loss while IP deficiency exacerbated RSV‐induced weight loss with delayed viral clearance and had greater IFN‐α and β protein expression post‐RSV challenge (Hashimoto et al., 2004; Toki et al., 2013). Secondly, like PGE_2_, PGI_2_–IP signalling also synergises with inflammatory cytokines (e.g., TNF‐α and IL‐1β)‐activated NF‐κB for production of IL‐6 in stromal cells (Honda, Segi‐Nishida, Miyachi, & Narumiya, 2006). However, opposite to the effects of PGE_2_, PGI_2_ also appeared to down‐regulate TNF‐α and CCL2 and thus to reduce recruitment of CCR2‐expressing monocytes and macrophages to inflammation sites (Kumei et al., 2018). Thirdly, PGI_2_ modulated adaptive T cell responses, for example, promoting inflammatory IFN‐γ‐producing Th1 and IL‐17‐producing Th17 cells (Nakajima et al., 2010; Truchetet, Allanore, Montanari, Chizzolini, & Brembilla, 2012; W. Zhou et al., 2012). Lastly, PGI_2_ stimulated Ca^2+^ efflux through IP‐activated cAMP, counteracting platelet activation (e.g., induced by TXA_2_), which is also akin to effects of the PGE_2_– EP4 receptor pathway. The ability of PGI_2_ to regulate immune and inflammatory responses thus has implications for COVID‐19.

### 4.6 Potential effects of clinical interventions of COVID‐19 on PG biosynthesis

There are many therapeutic strategies to combat COVID‐19 that have been attempted in a number of clinical trials. The main targets include (1) SARS‐CoV‐2 attachment and entry to human cells (e.g., the TMPRSS2 inhibitor camostat mesylate and viral fusion inhibitors such as hydroxychloroquine) and viral replication (e.g., remdesivir and lopinavir); (2) systemic immune responses, for example, biological agents, such as anti‐IL‐6, anti‐TNF, anti‐IL‐1β, anti‐IFN‐γ, or low MW inhibitors; and (3) hyperinflammation such as steroids,such as dexamethasone and NSAIDs. Trials using antibodies targeting cytokines or chemokines aim to block inflammatory immune cell migration and function and subsequently to reduce the cytokine storm‐induced systemic inflammation. As a result, down‐regulation of inducible COX‐2 and mPGES‐1 as well as PG biosynthesis is also expected, as discussed above. Apart from the small pilot trial administering celecoxib to COVID‐19 patients (Hong et al., 2020), another clinical trial has been registered as of July 9, 2020, proposing to use naproxen in critically ill COVID‐19 patients (https://clinicaltrials.gov/ct2/show/NCT04325633). As an inhibitor of both COX‐2 and IAV nucleoprotein, naproxen reduced mortality of patients with H3N2 influenza infection when administered in combination with clarithromycin and oseltamivir in a recent clinical trial (Hung et al., 2017). It is thus expected that naproxen may reduce the mortality associated with critical COVID‐19 patients via both COX‐independent antiviral transcription/replication and COX‐2‐dependent anti‐inflammatory effects (https://clinicaltrials.gov/ct2/show/NCT04325633). Although corticosteroids are not recommended for treatment of SARS (Stockman, Bellamy, & Garner, 2006), there are many clinical trials of steroids in COVID‐19 patients (https://clinicaltrials.gov). Strikingly, a very recent large‐scale clinical trial showed that low‐dose dexamethasone (6 mg once daily) reduced deaths by one third in ventilated patients with COVID‐19 and by one fifth in other COVID‐19 patients who received oxygen only, while no benefit was observed in COVID‐19 patients not requiring respiratory support (Horby et al., 2020). Given that corticosteroids including dexamethasone inhibit PG (especially PGE_2_) production from various human tissues such as gut and lung (Aksoy, Li, Borenstein, Yi, & Kelsen, 1999; Hawkey & Truelove, 1981; Ogushi, Ozaki, Kawano, & Yasuoka, 1987), results from this clinical trial are promising for COVID‐19 therapeutic strategies targeting PG pathways. Corticosteroids suppress PG production through inhibition of the enzymic activities of PLA_2_ and COX‐2, but not COX‐1 (M.‐Z. Zhang, Harris, & McKanna, 1999). Dexamethasone represses COX‐2 expression by both transcriptional and post‐transcriptional mechanisms; that is, dexamethasone reduces COX‐2 gene expression through thyroid hormone receptors interfering the binding of NF‐κB to the COX‐2 gene promoter and decreases COX‐2 mRNA stability via mechanisms involving preferential loss of polyadenylated mRNA, resulting in switching off mRNA translation and protein synthesis (Barnes, 2006; Newton, Seybold, Kuitert, Bergmann, & Barnes, 1998). Of note, despite inhibition of COX‐2 by both NSAIDs and steroids, they have different in vivo actions in managing inflammatory diseases. For example, while dexamethasone only had moderate effects on reducing mortality of severe/critical (but not non‐severe) COVID‐19 patients (Horby et al., 2020), celecoxib reduced disease severity and improved remission in both non‐severe and severe COVID‐19 patients (Hong et al., 2020), indicating different clinical outcomes from use of NSAIDs and other anti‐inflammatory agents. Nevertheless, further studies are essential to clarify the clinical safety and efficiency of these two families of anti‐inflammatory drugs and to understand the underlying immunomodulatory mechanisms.

## 5 CONCLUSIONS

**FIGURE 4:**
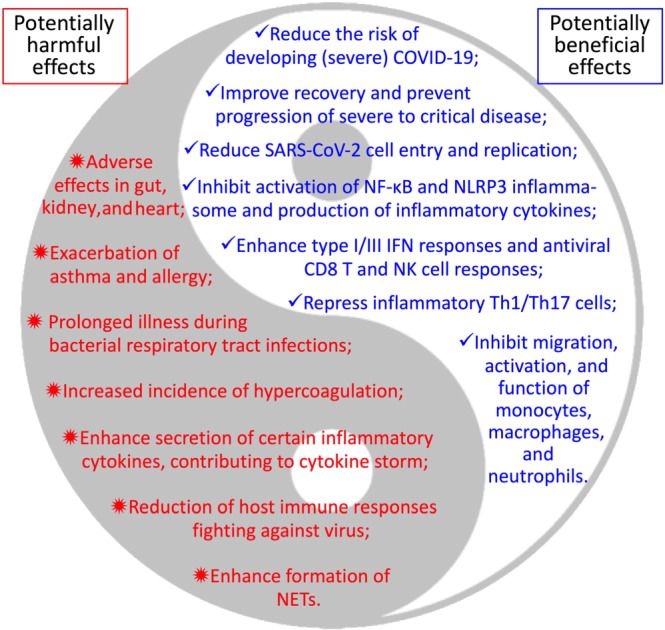
Yin and Yang of NSAIDs in COVID‐19: potential effects. A diagram showing potentially positive and negative effects of NSAID use in COVID‐19 patients

Here, we have discussed the potential actions of PGs in SARS‐CoV‐2 infection and COVID‐19 pathogenesis based on the analysis of how PGs work in other inflammatory conditions and infections, such as SARS‐CoV‐1 and MERS‐CoV, including the potential positive and negative effects of NSAIDs during COVID‐19 (Figure 4). While concerns were raised for ibuprofen use in COVID‐19 patients, epidemiological studies have suggested that benefits for NSAID use are likely, by reducing the risk of development of severe disease in COVID‐19 patients. This was supported by a pilot experimental trial in which celecoxib reversed COVID‐19 disease development and progression to a severe state. Further epidemiological analyses and large‐scale clinical trials are needed to clarify the effects of NSAIDs (and their associated risks) during SARS‐CoV‐2 infection and COVID‐19 disease severity. Substantial evidence suggests that PGs are likely to be involved in many stages of SARS‐CoV‐2 infections and the development of COVID‐19, although their actual role(s) remain to be elucidated. For example, PGs and their precursors may affect SARS‐CoV‐2 attachment by modulating ACE2 and TMPRSS2 expression and virus endocytosis by regulating lipid vesicle fusion. Importantly, PGs are more likely to modulate host immune and inflammatory responses activated by virus pathogens. PGs, especially PGE_2_, can foster or restrain both innate and adaptive immune reactions. For example, PGE_2_ can repress type I IFN signalling, cytotoxic T cell responses, NET formation, inflammasome activation, and inflammatory cytokine production, whereas it can also contribute to inflammatory Th17 responses, NF‐κB activation, and related inflammatory cytokine production. These effects of PGs rely, to a great extent, on the context and micro‐environments such as strength (e.g., viral load) and timing of stimuli, organ locations, and responding cell types. Given the critical role of cytokine storms in COVID‐19 immunopathology and the context‐dependent regulation of cytokine production by PGs, it is imperative to understand the chief cellular sources of cytokines in the lung and peripheral blood, the key stimuli (e.g., SARS‐CoV proteins or peptides) and to elucidate the kinetics of cytokine secretion. Results from such studies may be insightful for considerations on when exactly NSAIDs could be administered to COVID‐19 patients to tackle hyperinflammation while minimising their adverse side effects. Furthermore, as NSAIDs non‐selectively block all PG pathways by targeting COXs (that may be related to unfavourable side effects), targeting respective PG synthesis or PG receptors may help to avoid some of the adverse side effects of NSAIDs. By and large, current evidence is insufficient to support if PGs or, in other words, NSAIDs have beneficial or deleterious actions on SARS‐CoV‐2 infection, development, and disease progression, although PGE_2_ (maybe also PGD_2_) is likely to be involved in inhibiting host antiviral responses and development of hyperinflammation in patients with COVID‐19. In light of the evidence detailed in this review, it is imperative to obtain comprehensive understanding of which immune response(s) could predominate at each stage of COVID‐19, and how PGs modulate different cell type‐dependent anti‐inflammatory and proinflammatory responses during the course of COVID‐19. Such insights will improve clinical understanding, especially in attempting to decide when and what specific NSAID could be administered at what dose ranges during disease progression in COVID‐19 patients, taking into careful consideration any co‐morbidities, as well as the known side effects of NSAIDs.

### 5.1 Nomenclature of targets and ligands

Key protein targets and ligands in this article are hyperlinked to corresponding entries in http://www.guidetopharmacology.org, the common portal for data from the IUPHAR/BPS Guide to PHARMACOLOGY (Harding et al., 2018), and are permanently archived in the Concise Guide to PHARMACOLOGY 2019/20 (Alexander, Christopoulos, et al., 2019; Alexander, Fabbro, et al., 2019a, b; Alexander, Kelly, et al., 2019).

## CONFLICT OF INTEREST

The authors declare no conflicts of interest.
